# Distinct genetic clusters in HIV-1 CRF01_AE-infected patients induced variable degrees of CD4^+^ T-cell loss

**DOI:** 10.1128/mbio.03349-23

**Published:** 2024-02-22

**Authors:** Kang Li, Huanhuan Chen, Jianjun Li, Yi Feng, Shujia Liang, Abdur Rashid, Meiliang Liu, Sisi Li, Qingfei Chu, Yuhua Ruan, Hui Xing, Guanghua Lan, Wentao Qiao, Yiming Shao

**Affiliations:** 1 Key Laboratory of Molecular Microbiology and Technology, Ministry of Education, College of Life Sciences, Nankai University, Tianjin, China; 2 National Key Laboratory of Intelligent Tracking and Forecasting for Infectious Diseases, National Center for AIDS/STD Control and Prevention, Chinese Center for Disease Control and Prevention, Beijing, China; 3 Guangxi Key Laboratory of Major Infectious Disease Prevention Control and Biosafety Emergency Response, Guangxi Center for Disease Control and Prevention, Nanning, China; 4 School of Medicine, Nankai University, Tianjin, China; 5 School of Public Health, Guangxi Medical University, Nanning, Guangxi, China; 6 School of Medicine, Zhejiang University, Hangzhou, China; 7 Changping Laboratory, Beijing, China; The University of North Carolina at Chapel Hill School of Medicine, Chapel Hill, North Carolina, USA

**Keywords:** HIV-1, genetic cluster, viral virulence, co-receptor tropism, CD4^+^ T-cell count

## Abstract

CRF01_AE strains have been shown to form multiple transmission clusters in China, and some clusters have disparate pathogenicity in Chinese men who have sex with men. This study focused on other CRF01_AE clusters prevalent in heterosexual populations. The CD4^+^ T-cell counts from both cross-section data in National HIV Molecular Epidemiology Survey and seropositive cohort data were used to evaluate the pathogenicity of the CRF01_AE clusters and other HIV-1 sub-types. Their mechanisms of pathogenicity were evaluated by co-receptor tropisms, predicted by genotyping and confirmed with virus isolate phenotyping, as well as inflammation parameters. Our research elucidated that individuals infected with CRF01_AE clusters 1 and 2 exhibited significantly lower baseline CD4^+^ T-cell counts and greater CD4^+^ T-cell loss in cohort follow-up, compared with other HIV-1 sub-types and CRF01_AE clusters. The increased pathogenesis of cluster 1 or 2 was associated with higher CXCR4 tropisms, higher inflammation/immune activation, and increased pyroptosis. The protein structure modeling analysis revealed that the envelope V3 loop of clusters 1 and 2 viruses is favorable for CXCR4 co-receptor usage. Imbedded with the most mutating reverse transcriptase, HIV-1 is one of the most variable viruses. CRF01_AE clusters 1 and 2 have been found to have evolved into more virulent strains in regions with predominant heterosexual infections. The virulent strains increased the pressure for early diagnosis and treatment in HIV patients. To save more lives, HIV-1 surveillance systems should be upgraded from serology and genotyping to phenotyping, which could support precision interventions for those infected by virulent viruses.

**IMPORTANCE:**

Retroviruses swiftly adapt, employing error-prone enzymes for genetic and phenotypic evolution, optimizing survival strategies, and enhancing virulence levels. HIV-1 CRF01_AE has persistently undergone adaptive selection, and cluster 1 and 2 infections display lower counts and fast loss of CD4^+^ T cells than other HIV-1 sub-types and CRF01_AE clusters. Its mechanisms are associated with increased CXCR4 tropism due to an envelope structure change favoring a tropism shift from CCR5 to CXCR4, thereby shaping viral phenotype features and impacting pathogenicity. This underscores the significance of consistently monitoring HIV-1 genetic evolution and phenotypic transfer to see whether selection bias across risk groups alters the delicate balance of transmissible versus toxic trade-offs, since virulent strains such as CRF01_AE clusters 1 and 2 could seriously compromise the efficacy of antiviral treatment. Only through such early warning and diagnostic services can precise antiviral treatments be administered to those infected with more virulent HIV-1 strains.

## INTRODUCTION

RNA viruses are adaptable as a result of frequent evolutionary changes, thus allowing them to adopt effective survival strategies and maintain a certain level of virulence. Specifically, human immunodeficiency virus type 1 (HIV-1) exhibits a heightened mutation rate and has evolved into multiple sub-types and circulating recombinant forms (CRFs) during the global transmission process (1). These variants demonstrate diverse transmission routes, geographic distributions, and prevalence patterns (2). Noteworthy among them is CRF01_AE, a prominent CRF that exhibits global prevalence with a particular emphasis in Southeast and East Asia (3, 4). The extensive and rapid dissemination of the CRF01_AE strain plays a critical role in the diversity and complexity of HIV-1 infection in China, based on the National HIV Molecular Epidemiology Survey (5). Moreover, CRF01_AE, subjected to years of adaptive selection within diverse high-risk cohorts, has progressively formed several stable transmission clusters, potentially characterized by varying degrees of virulence and rates of disease progression (6–9).

Extensive literature highlights significant differences in viral virulence among HIV sub-types, including CRF01_AE (10–13). Notably, CRF01_AE infections are associated with faster CD4 cell loss and shorter overall survival compared to other sub-types (8, 14–17). The current consensus posits that the type of co-receptor binding to the cell is of paramount importance in influencing virulence, commonly serving as the mechanism for variations in virulence between HIV sub-types or CRFs (18, 19). Earlier studies have shown that CRF01_AE-infected individuals typically exhibit a higher frequency of CXCR4 tropic virus infection (9, 20–22). Additionally, distinctions have been observed within CRF01_AE transmission clusters, specifically in men who have sex with men (MSM) clusters 4 and 5(13). However, considering the prevalence of CRF01_AE HIV-1 in China, studies show that each cluster seems to play a distinct role, exhibiting varying associations with specific risk groups and geographic regions (4). The current hypothesis posits that the heterogeneity within CRF01_AE clusters may contribute significantly to unique phenotypic expressions and pathogenicity, a causation attributed to the genetic determinants of the virus.

Measuring CD4^+^ T cells in peripheral blood is commonly used to assess HIV-1 disease progression. In this study, we systematically analyzed the trajectories of CD4^+^ T-cell counts within CRF01_AE clusters in the National HIV Molecular Epidemiology Survey (NHMES) database, adjusting for infection timelines to assess the viral impact on the immune system. Simultaneously, we observed key phenotypic trends and heritable properties driving changes in CRF01_AE variant virulence. It was revealed that pathogenesis of the CRF01_AE variants was incredibly diversified; clusters 1 and 2 exhibit earlier CXCR4 co-receptor tropism shift due to genetic fitness, protein modifications, and structural features, which contribute to the exceptionally high virulence of these clusters compared with other CRF01_AE clusters and CRF07 and 08_BC.

## RESULTS

### CD4^+^ T-cell counts and their relative reduction in individuals infected with different CRF01_AE clusters

**Fig 1:**
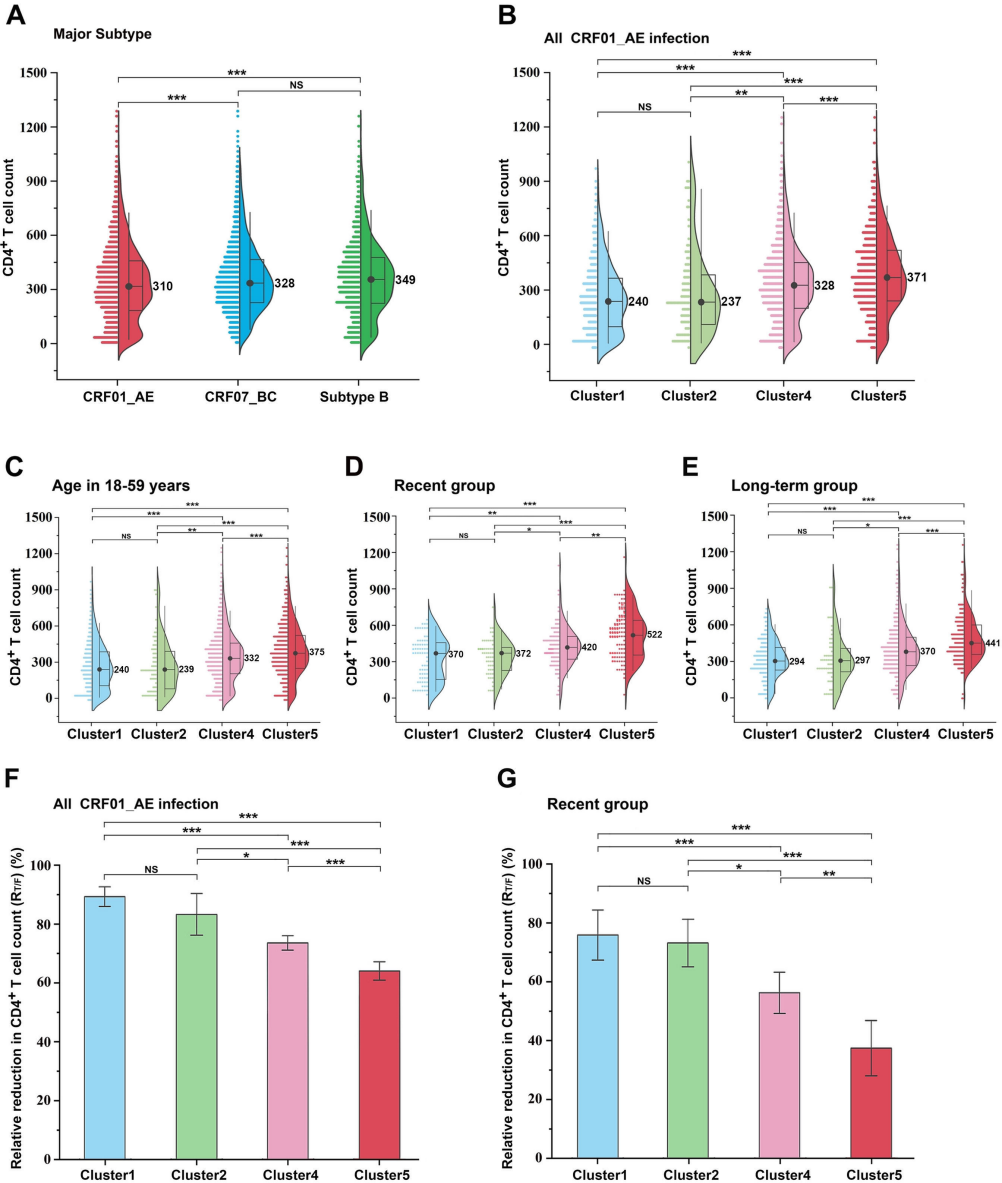
Comparative evaluation of baseline CD4^+^ T cells and their reduction in HIV-1 sub-types and CRF01_AE clusters. (**A**) Differences in CD4^+^ T-cell count were assessed across serum samples from individuals infected with CRF01_AE (*n* = 2,683), CRF07_BC (*n* = 3,347), and sub-type B (*n* = 1,166) based on data from the National HIV Molecular Epidemiology Survey. (**B**) Comparison of CD4^+^ T-cell counts among different CRF01_AE clusters, including cluster 1 (*n* = 469), cluster 2 (*n* = 131), cluster 4 (*n* = 1,174), and cluste r5 (*n* = 773). (**C**) Comparison of CD4^+^ T-cell counts among different CRF01_AE clusters based on individuals in the age group of 18–59 years. (**D**) Comparison of CD4^+^ T-cell counts among different CRF01_AE clusters in the recent infection group. (**E**) Comparison of CD4^+^ T-cell counts among different CRF01_AE clusters in the long-term infection group. (F and G) Relative reduction in CD4^+^ T-cell counts among different CRF01_AE clusters. The middle bars, box, and whiskers indicate the median, interquartile range, and the 5th–95th percentiles, respectively. The non-parametric two-tailed Mann-Whitney *U* test was used to determine statistical differences in CD4^+^ T-cell count. The *t*-test was used to determine the mean *R*_T/F_ difference in CRF01_AE clusters. *P < 0.05, **P < 0.01, ***P < 0.001. NS, not statistically significant.

Herein, we assessed absolute CD4^+^ T-cell counts in three sets of HIV variants. At the time of diagnosis, CD4^+^ T-cell counts for 2,683 CRF01_AE-infected individuals were lower than those for 3,347 CRF07_BC-infected and 1,166 sub-type B-infected individuals (Fig. 1A). In a preliminary study, we identified at least five genetic sub-clusters of CRE01_AE with different prevalence patterns (4). When comparing the four major clusters of CRF01_AE independently, which contribute significantly to the prevalence of heterosexual transmission (HET) and MSM in China. Individuals infected with clusters 1 and 2 exhibited considerably lower CD4^+^ T-cell counts than those infected with clusters 4 and 5, although lower CD4^+^ T-cell counts were also found in cluster 4 patients compared to cluster 5 patients (Fig. 1B). Then, to determine whether age influenced this trend, patients were classified into 18–59 age groups. Figure 1C shows that CRF01_AE clusters 1 and 2 still had lower CD4^+^ T-cell counts. As CD4^+^ T-cell counts decreased over time post-spontaneous infection, patients were classified into recent infection and long-term infection groups based on HIV-1 restriction antigen effectiveness testing. Baseline CD4^+^ T-cell counts were significantly lower in patients infected with clusters 1 and 2 compared to those infected with clusters 4 and 5 in both groups (Fig. 1D and E). Moreover, cluster 4-infected patients had lower CD4^+^ T-cell counts compared with those infected with cluster 5. Overall, the data clearly reveal that the CRF01 AE clusters were different from each other; clusters 1, 2, and 4 resulted in significantly lower CD4^+^ T-cell counts during early infection.

*R*_T/F_ values were used as a metric to calculate the relative decrease in CD4^+^ T-cell count associated with HIV-1 infection. In Fig. 1F, *R*_T/F_ values of CRF01_AE clusters 1 and 2 were 89.3% and 83.5%, respectively, which were higher than those of clusters 4 and 5 (73.5% and 64.5%, respectively) (*P* < 0.05). A similar trend was observed in recent HIV infections, with *R*_T/F_ values of clusters 1 and 2 (76.0% and 73.5%, respectively) considerably higher than those of clusters 4 and 5 (56.7% and 38.4%, respectively; *P* < 0.05; Fig. 1G). Collectively, comparing *R*_T/F_ values revealed higher variations among CRF01_AE sub-clusters than comparing absolute CD4^+^ T-cell counts.

### CXCR4 usage varied among CRF01_AE clusters

CXCR4-tropic viruses are intricately linked to diminished CD4^+^ T-cell counts and the progression of the disease. Here, genotypic prediction was used to calculate the CXCR4/CCR5-CXCR4-tropic virus ratio among CRF01_AE different clusters. We observed a higher prevalence of the CXCR4 phenotype in patients with CD4^+^ T-cell counts below 200 (Table S1). In comparison to other clusters, patients in clusters 1 and 2 exhibited a higher proportion of individuals with CD4^+^ T-cell counts below 200 (Table S2). Overall, significant differences in the frequency of occurrence of CXCR4/CCR5-CXCR4 viruses were predicted in the CRF01_AE internal cluster, with cluster 1 (22.1%) and cluster 2 (20.5%) having higher frequencies compared to cluster 4 (15.2%) and cluster 5 (6.8%) (Table S3). This trend implied that differences in CD4^+^ T-cell counts among CRF01_AE sub-clusters were substantially linked to differences in CXCR4 prevalence rates.

### CXCR4 tropism among recently infected patients

**Fig 2:**
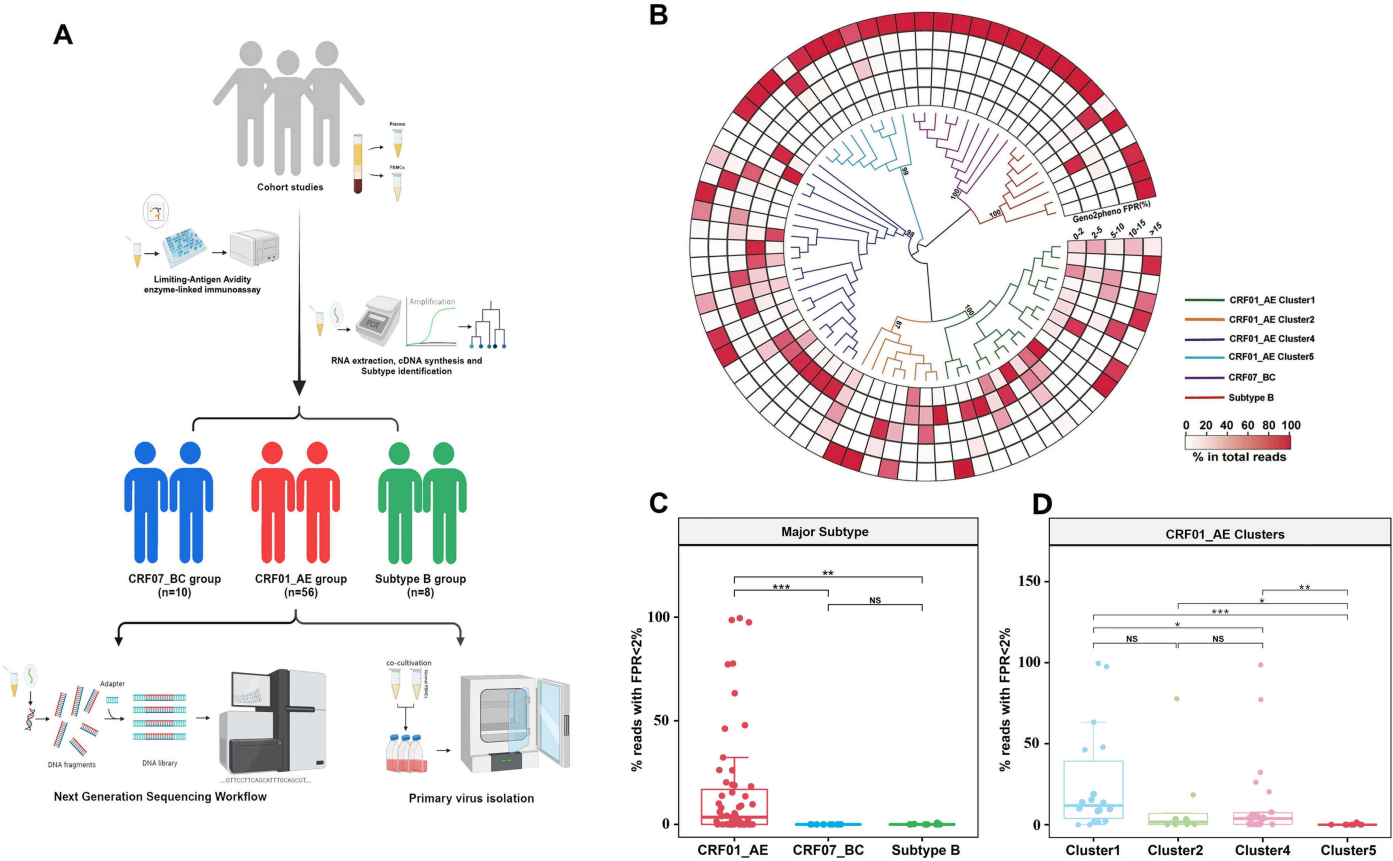
Comparison of the predicted frequency of X-using strains among different HIV-1 sub-types and CRF01_AE clusters by next-generation sequencing. (**A**) Overview of the study participants. In the initial sample set; a total of 74 samples successfully met the screening criteria. This subset comprised 56 samples with pure CRF01_AE, 10 samples with CRF07_BC, and 8 samples with B sub-type infections. Next-generation sequencing was performed on plasma samples obtained from this selected population, and their peripheral blood mononuclear cells (PBMCs) underwent primary virus isolation. (**B**) Phylogenetic relationship of recently infected individuals in two cohort studies, including those infected with CRF01_AE clusters, CRF07_BC, and sub-type B viruses. The maximum likelihood tree was constructed using the most frequent haplotype sequences under the GTR + I + G site substitution model. Different colors represent sub-type sequences. (**C and D**) Heatmap depicting the frequency distribution of Geno2-pheno predicted false-positive rate (FPR) values in next-generation sequencing reads for each sample. The proportion of next-generation sequencing reads with Geno2pheno FPR of <2% in each sample was determined. Each dot indicates the frequency from a single individual. Two-tailed Mann-Whitney *U* test was employed to determine the statistical difference. ***P < 0.001, *P < 0.05, **P < 0.01, ***P < 0.001. NS, not statistically significant.

Subsequently, we performed next-generation sequencing on recently HIV-infected individuals in the two cohort studies infected by CRF01_AE, CRF07_BC, and sub-type B to determine whether differences in CD4 counts among CRF01_AE sub-clusters could be attributed to high CXCR4 tropism (Fig. 2A). In patients recently infected with CRF01_AE, the numbers of patients infected with cluster 1, 2, 4, and 5 were 18, 8, 21, and 9, respectively (Fig. S1). A total of 55,000 sequences were obtained on average per participant, and genotypes were predicted using Geno2pheno (Fig. 2B; Tables S4 and S5). Based on false-positive rate (FPR) distribution in viral quasi-species in individual plasma samples, the frequency of predicted CXCR4 virus in the CRF01_AE was higher than that in the non-CRF01_AE sub-types (such as sub-type B or CRF07_BC) (Fig. 2C, *P* < 0.001). Moreover, CXCR4 tropism was significantly more prevalent in CRF01_AE clusters 1 and 2 than in clusters 4 and 5 (Fig. 2D, *P* < 0.05).

### CXCR4 tropism was associated with low CD4^+^ T-cell counts

**Fig 3:**
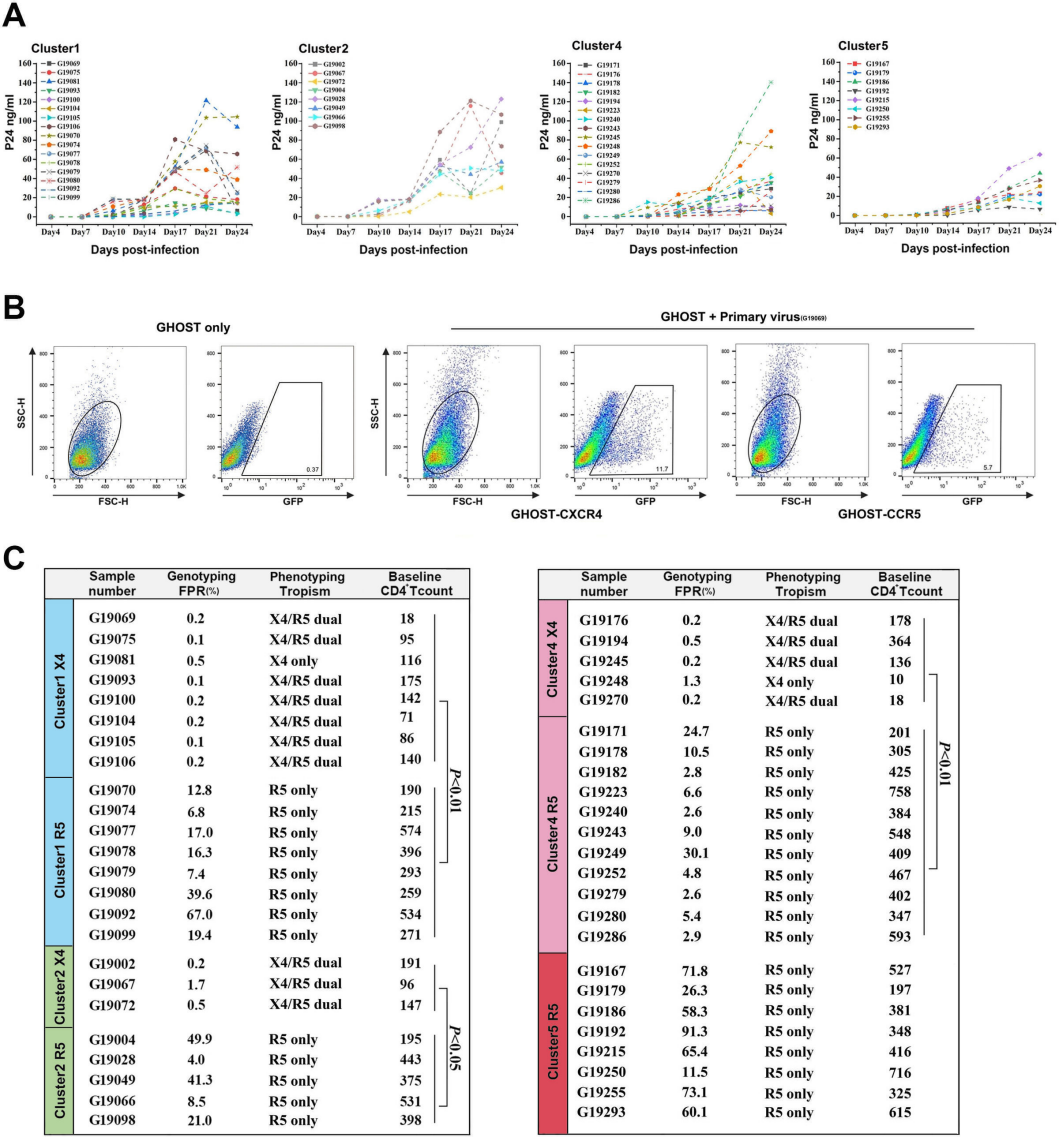
HIV-1 isolation and phenotyping their co-receptor tropisms. (**A**) Replication dynamics of HIV strains isolated from infected peripheral blood mononuclear cells of patients infected with CRF01_AE clusters 1, 2, 4, and 5. P24 antigen levels in the supernatant of duplicate samples were measured to monitor viral replication. (**B**) Expression of green fluorescent protein (GFP) in GHOST.CXCR4 cells and GHOST.CCR5 cells, indicating the tropism of primary viral isolates in samples. (**C**) The baseline CD4^+^ T-cell counts of participants and false-positive rate (FPR) values from the Geno2Pheno method for predicting co-receptor usage. The comparison of CD4^+^ T-cell counts in individuals infected with CXCR4 and CCR5 viral phenotypes was performed using the two-tailed Mann-Whitney *U* test.

We subsequently conducted phenotypic validation of the genetic predictions through the isolation of the virus from peripheral blood mononuclear cells (PBMCs) obtained from individuals belonging to the CRF01_AE clusters 1, 2, 4, and 5. A total of 48 distinct viral strains were successfully isolated from the PBMCs of individuals within the CRF01_AE cluster (Fig. 3A). In addition, 19 viral strains showed tropism to CCR5 only, while 16 viral strains were CXCR4/CCR5-tropic (Fig. 3B and C). Remarkably, cluster 5 viruses were isolated only with the CXCR5 tropical phenotype. Inhibition experiments with the CCR5 inhibitor Maraviroc and the CXCR4 inhibitor AMD3100 further revealed that G19081 and G19248 entered exclusively using CXCR4 (Fig. S2). This suggests a higher prevalence of CXCR4 tropic viruses in CRF01_AE clusters 1, 2, and 4 compared to cluster 5, which is consistent with genotype prediction. Among individuals infected with the verified viral phenotype, CD4^+^ T-cell counts were significantly higher in CCR5-tropic viruses than in the CXCR4-tropic phenotype (Fig. 3C). These phenotypic data highlight the association between CXCR4 usage and lower CD4^+^ T-cell counts, prompting further investigation into the underlying sequence-based mechanisms of CXCR4 receptor selection.

### Genetic and structural properties of CXCR4 co-receptor usage

**Fig 4:**
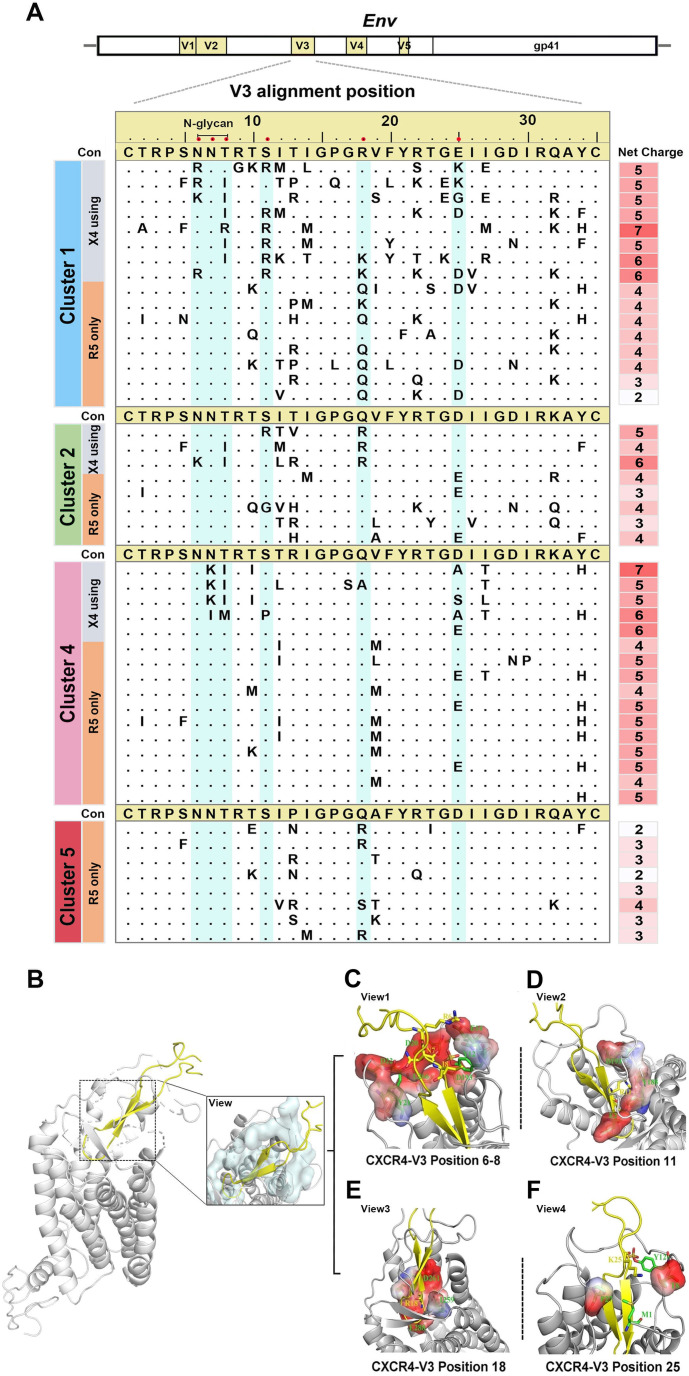
Genetic features of CXCR4 sequences with phenotypic confirmation and structural analyses of co-receptor binding models. (**A**) Amino acid positions and net charge distribution in the V3 region of phenotype-confirmed primary viral isolates with CXCR4 usage. Crucial CXCR4 utilization-associated V3 positions are highlighted in cyan. (**B**) Structural modeling of the binding site between the co-receptor CXCR4 and the V3 region using the docking model. The interfaces between co-receptor CXCR4 and V3 are depicted with an overall side view and detailed in a close-up view (right boxed). (**C–F**) Detailed description of the interactions with the major sites of the V3 loop in the CXCR4 binding pocket. The surface of CXCR4 binding to V3 is displayed with negative (red colored) and positive (blue colored) electrostatic potentials. Key residues are depicted as sticks, and the overall structure is shown as a graphical representation.

Because the *env*-V3 loop of the virus plays a crucial role in binding to the target cell co-receptor, we analyzed the *env*-V3 loop sequence properties of CXCR4 and CCR5 viruses in clusters 1 and 2 to elucidate the molecular determinants for co-receptor switching. Unlike CCR5, most cluster 1 and 2 CXCR4 sequences lacked the N-linked glycosylation site at the beginning of the V3 loop (positions 6–8). Basic arginine (R)/lysine (K) and isoleucine (I) were more frequent in positions 6 and 8, respectively (Fig. 4A). Residual asparagine (N) was retained in the seventh position in all sequences, which was consistent with the CCR5 sequence. Cluster 4 CXCR4 sequences commonly exhibited N-chain glycosylation site deletions at positions 7 and 8. Combination substitutions, including serine (S) 11 and/or glutamine (Q) 18, and at least one basic amino acid residue (R/K), were more common in CXCR4 than in CCR5 sequences (Fig. 4A). CCR5 sequences exhibited glutamic (E) or aspartic (D) residues stably at position 25, with only cluster 1 CXCR4 sequence showing K substitutions (Fig. 4A). Notably, cluster 4 CXCR4 sequences lacked basic amino acids R or K at both positions 11 and 25 of the V3 loop. This observation implies distinct evolutionary mechanisms for co-receptor switching within the CRF01_AE sub-clusters.

To further broaden our understanding of the specific sites within the V3 loop, we subsequently simulated the docking of the CXCR4 co-receptor with the V3 loop. Following extensive optimization between interaction interfaces and pocket structures, we were able to reconstruct the protein structure diagram of the CXCR4-V3 (CRF01_AE) complex (Fig. 4B). We observed that negatively charged residues were aligned near the V3 N-linked glycosylation site (positions 6–8), thus facilitating the interaction of positively charged residues (R or K) in the sequence with the CXCR4 receptor (Fig. 4C). As shown above, the residue I8 in the loop region was surrounded by hydrophobic residues, which could explain the substitution of T to I at position 8 in CXCR4 sequences, with residue T8 unsuitable for establishing a lateral hydrophobic environment in binding interactions. Meanwhile, residues at positions 11 and 18 within the CXCR4 sequence were also negatively charged around the ligand-binding interface, thereby contributing to the preferential occurrence of positively charged residue sites (R or K) in the virus (Fig. 4D and E). Further structural analysis revealed that residue R11 in CXCR4 virus may form salt bridges with glutamic (E)2, tyrosine (Y)188, and aspartic (D)193 residues, while R18 may form hydrogen bonds with I259, D261, and E288 in the CXCR4 co-receptor. Moreover, position D/E25 showed less preference due to a less positively charged interaction environment at the docking site. K substitutions in CXCR4 allowed the residue K25 in the virus to form salt bridges with the N terminus of CXCR4 for efficient binding (Fig. 4F). Overall, the specific structural and interaction environment of the V3-loop sites may be critical for the generation of the CXCR4 phenotype in CRF01_AE clusters 1 and 2, which differed from the core locations, such as 13R and 32K, described in cluster 4 and 5 (13).

### Evolutionary dynamics and conservation of important sites involved in CXCR4 co-receptor usage

**Fig 5:**
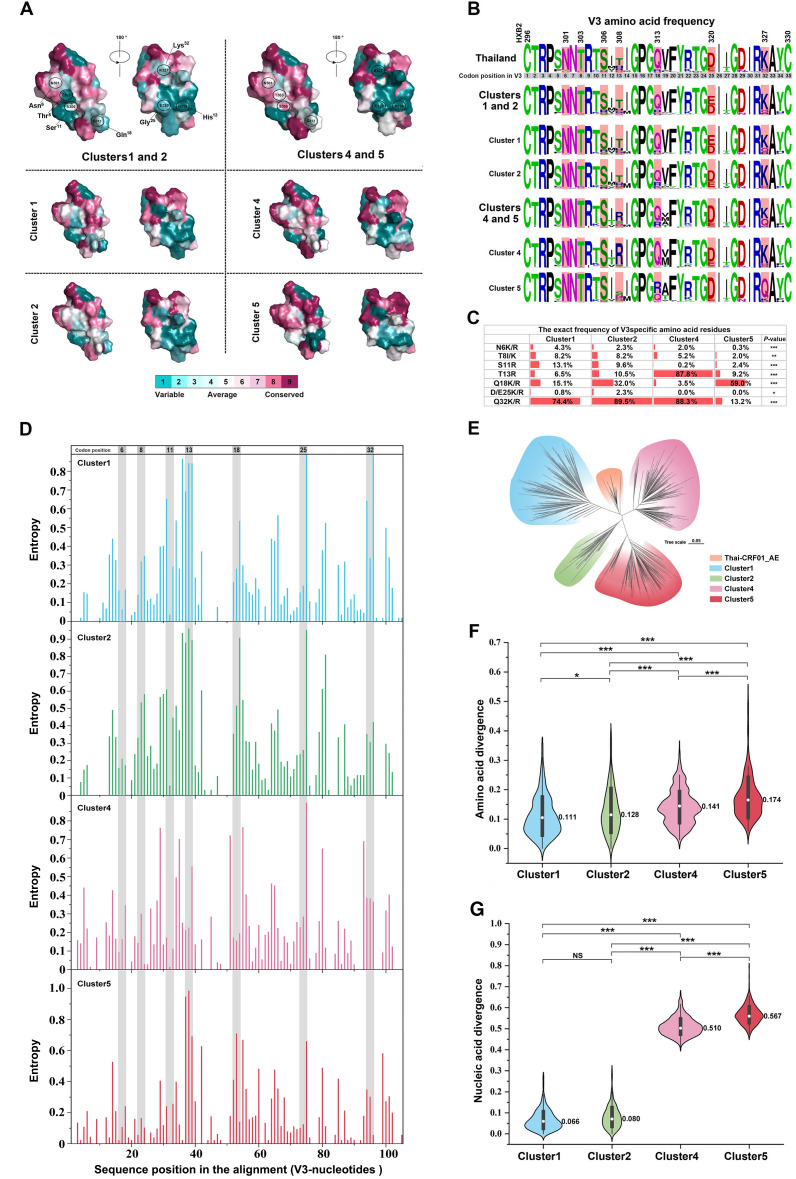
Conserved amino acid sites in the V3 region on the surface of the CRF01_AE clusters. (**A**) Surface depiction of conserved V3 amino acid residues in clusters 1 (*n* = 521), 2 (*n* = 249), 4 (*n* = 597), and 5 (*n* = 341), colored from variable (teal) to conserved (fuchsia) as determined by Consurf analysis based on the available sequences (NHMES). The color gradient represents the level of sequence conservation determined based on the Consurf database. (**B**) Frequency of amino acids at key V3 positions related to the CXCR4-using phenotype in each cluster. Sequences from Thailand (*n* = 93) were obtained from the Los Alamos HIV sequence database (prior to the year 1999). (**C**)The exact frequencies are provided at the bottom. Differences in proportions or rates between clusters 1, 2 , 4, and 5 are assessed using the Cochran-Armitage trend test. (**D**) ENTROPY calculates the entropy of each position in an input sequence set. It is used to measure the relative variability of different positions or regions within the V3 loop gene of each cluster. The vertical axis signifies entropy scores, delineating the level of variability, while the horizontal axis denotes the position of amino acids within the *env*-V3 gene. (**E**) Based on a phylogenetic analysis of distinct clusters in China and early-stage CRF01_AE sub-type in Thailand (prior to the year 1999), an unrooted phylogenetic tree was constructed using sequences from the *env* C2-C4 region. (**F** and G) Nucleic acid and amino acid divergence in the V3 region for clusters 1, 2, 4 and 5 compared to the Thailand consensus sequence (generated from 93 sequences). Statistical significance is determined by one-way analysis of variance. *P < 0.05, **P < 0.01, ***P < 0.001. NS, not statistically significant.

To understand the structurally conserved characteristics of CXCR4 usage among CRF01_AE subgroups, we analyzed sequence conservation of four cluster-related V3 proteins from the NHMES. The N-linked glycosylation site (HXB2 positions N301–T303), as well as positions H308, K327, and E320, exhibited higher variability. Clusters 1 and 2 had discrete patches in the variable region N301–T303, directly affecting CXCR4. The structural motifs at location S306, which were in contact with CXCR4 binding planes, were conserved in clusters 4 and 5 (Fig. 5A). Subsequently, the frequency of V3 amino acids among clusters was evaluated (Fig. 5B and C). Within the highly variable area, a higher frequency of basic amino acid residues N6K/R, T8I/K, S11R, D/E25K/R, and Q32K/R was found in clusters 1 and 2 compared to clusters 4 and 5 (*P* < 0.05), while relatively lower-frequency mutations were found in T13R (*P* < 0.05). Of particular interest, position Q18K/R displayed distinct mutation patterns. Clusters 1, 2, and 5 demonstrated markedly elevated mutation rates at this position relative to cluster 4. Concurrently, each column in the alignment of V3 nucleotide sequences underwent independent analysis, with entropy scores assigned to delineate the variability of specific positions. The outcomes revealed distinct variabilities at these specific positions across clusters, particularly showcasing heightened instability at critical points within clusters 1 and 2, notably at positions 8, 11, and 13, as compared to clusters 4 and 5. Notably, in contrast to clusters 1, 2, and 4, the virus within cluster 5 manifested overall stability, with crucial viral positions maintaining a stable state (Fig. 5D).

**Fig 6:**
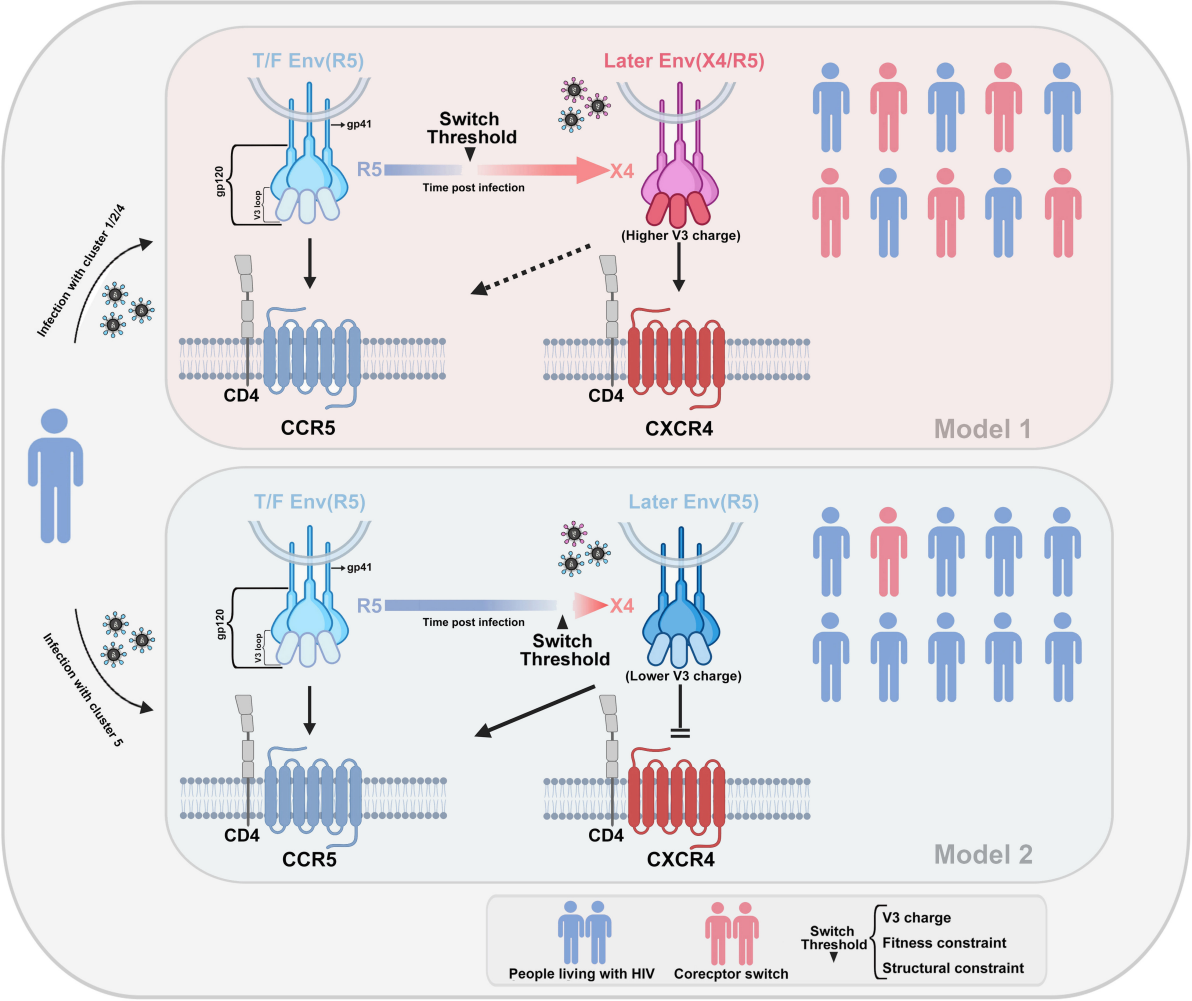
Schematic representation of the molecular model of the CD4-dependent HIV co-receptor tropism switch. The co-receptor tropism of HIV is determined by the sequence of the V3 structural domain. The envelope protein gp120 of HIV interacts with the CD4^+^ T receptor, leading to a conformational change in the V3 loop. Depending on the amino acid sequence and structural characteristics of V3, the virus can bind to either CCR5 or CXCR4 co-receptors. Certain sub-types of viruses have a low threshold for co-receptor switching to CXCR4 tropism due to the net charge number and structural characteristics of the V3 loop. Other viruses possess a high conversion threshold and maintain CCR5 tropism, interacting with the CCR5 co-receptor over an extended period.

We also assembled C2–C4 region genes from diverse clusters, as their ancestral strains originated from CRF01_AE in Thailand. To observe the consistency and divergence in genetic evolution, we particularly included previously reported strains from Thailand (4). Our phylogenetic analysis showed distinct differentiation within this gene region among various subgroups, each forming densely clusters (Fig. 5E). Regarding phylogenetic relationships with Thai strains, clusters 1 and 2 appeared closely related, suggesting the retention of more maternal genetic characteristics. Conversely, cluster 5 was significantly diverged, presenting unique features. Additionally, in terms of sequence conservation within the conserved region, clusters 1 and 2 exhibited a relatively smaller degree of divergence when compared to the CRF01_AE ancestor sequences from Thailand. In contrast, clusters 4 and 5 displayed a more substantial level of divergence, with cluster 5 showing a particularly pronounced increase in amino/nucleic acid divergence (Fig. 5F and G). Overall, these non-synonymous mutations conferred a more positive net charge in the V3 loop to cluster 1, 2, and 4 viruses. Thus, a highly variable core site and structural adaptations in the V3 loop may reduce the threshold for CCR5–CXCR4 conversion in clusters 1, 2, and 4 due to their higher net positive charge. However, viruses resembling cluster 5 exhibited a higher genetic and functional barrier to this conversion (Fig. 6).

### Immune activation and pyroptosis in HIV-1-infected individuals with lower CD4^+^ T-cell counts

HIV-1 infection leads to progressive CD4^+^ T-cell depletion linked to inflammation *in vivo* (23, 24). Apoptosis accounts for approximately 5% of CD4^+^ T-cell depletion, while the remaining 95% involves pyroptosis in quiescent CD4^+^ T cells (23, 25, 26). The CXCR4 genotype was shown to promote rapid disease progression, especially in individuals with lower CD4^+^ T-cell counts (Table S1). We further examined the expression of inflammasome genes in PBMCs to determine the level of pyroptosis in HIV-1-infected patients with low CD4^+^ T-cell counts. A significant upregulation of genes coding for NLRP3 (*P* < 0.05), caspase-1 (*P* < 0.05), and caspase-3 (*P* < 0.02) was found in individuals with lower CD4^+^ T-cell counts (≤200) compared to those with higher CD4^+^ T-cell counts (>200) (Fig. S3A through C). Notably, caspase-1 and NLRP3 gene activations are essential for the inflammasome pathway and pyroptotic cell death, respectively. Moreover, we investigated the expression levels of interleukin (IL)-1β and IL-18 in the plasma of two groups of individuals. Results showed that higher expression levels of genes coding for IL-1β and IL-18 were found in individuals with lower CD4^+^ T-cell counts (Fig. S3D and E). Both IL-1β and IL-18 release indicated significant negative correlations with CD4^+^ T-cell counts (Fig. S3F and G). Thus, these CXCR4-infected individuals had a persistent inflammatory environment *in vivo*, and cell-dependent pyroptosis also played an essential role in the loss of CD4^+^ T cells in the initial stages of infection.

## DISCUSSION

The properties associated with the evolution of viruses to greater virulence have been broadly investigated, with the most recent example being the many variants of SARS-CoV-2 with differential transmissibility and varying degrees of risk (27, 28). Previous surveys on the heritability of viral load and CD4^+^ T-cell level loss made us identify that these characteristics may be altered by the presence of different variants of CRF01_AE (29). Moreover, genetic differences among T/F strains in some clusters have been previously described (13), but their manifestation in the broader sub-cluster population remains unclear. Herein, a large-scale NHMES study was conducted and showed significant differences in baseline CD4^+^ T-cell counts in HIV-1-infected patients with CRF01_AE clusters 1, 2, 4, and 5, driven by differences in strain virulence both in the earliest and latest stages of HIV infection. Lower CD4^+^ T-cell counts in clusters 1 and 2 correlated with higher prevalence of CXCR4 viruses. Their mutations in the envelope region and structural characteristics may contribute to CXCR4 co-receptor selection preference and applicability. This also demonstrated that virulence is a feature of the viral cluster, not a confounding characteristic of infected individuals.

CCR5-tropic viruses are initially dominant in the earliest stages of AIDS both through mucosal and parenteral routes, followed by CXCR4 variants as immunodeficiency progresses. Nearly 50% of sub-type B-infected individuals undergo co-receptor switching within approximately 5 years of infection, leading to a rapid decline in CD4^+^ T-cell count and accelerating AIDS progression. Numerous studies have observed that individuals infected with the CRF01_AE sub-type in China exhibit a higher proportion of CXCR4-tropic or R5 × 4-tropic viruses compared to those infected with non-CRF01_AE (8, 14, 17). This phenomenon is associated with an earlier occurrence of co-receptor switch and a faster disease progression time frame, particularly within the internal clusters of the CRF01_AE sub-types, where substantial differences in the dynamics of co-receptor switching are evident. Overall, the co-receptor switch in CRF01_AE clusters 1 and 2 occurs much earlier compared to clusters 4 and 5, as well as other HIV-1 sub-types. The World Health Organization designates individuals with a CD4^+^ cell count of 200 cells/mm³ as “advanced HIV disease” (30). Therefore, without timely intervention, it is anticipated that patients in clusters 1 and 2 of CRF01_AE will progress to advanced HIV disease relatively quickly from the time of infection. In older populations, the pace of progression to advanced HIV infection accelerates further, significantly increasing the risk of severe AIDS-related events, including death. Consequently, many individuals in clusters 1 and 2 may be diagnosed in the late stages of AIDS, with a poorer prognosis despite treatment. Currently prevalent in MSM and HET populations, CRF01_AE, particularly clusters 1 and 2, is widely disseminated in the HET population, while clusters 4 and 5 predominate in MSM (4, 6). In practice, significant delays in the time from infection to the initiation of treatment still exist among different risk populations. Thus, even in populations or regions with high awareness and monitoring of HIV-1 epidemiology, the variations in the CRF01_AE cluster remain a noteworthy concern. CRF01_AE has different cell entry environments and immune pressures in different risk populations, together with the evolutionary potentiality of the virus itself, which results in distinct adaptive mutations in these virus clusters.

Co-receptor selection depends on properties of the V3 loop of gp120. A higher positive net charge in the V3 loop commonly leads to interaction with the CXCR4 co-receptor, whereas a neutral net charge leads to binding to the CCR5 co-receptor (31–33). The results of our study confirmed these features, with the CXCR4 receptor binding interface showing a more negative charge than the CCR5 binding site. Typically, amino acid substitutions with residues R or K at positions 11 and 25 confer the ability to use the CXCR4 receptor (rule 11/15). CXCR4 clusters 4 and 5 showed fewer R or K mutations at positions 11 and 25 of the V3 loop compared to clusters 1 and 2 viruses. Post-translational modification of the V3 loop also influences co-receptor usage. CCR5-tropic viruses are more frequently glycosylated, thus carrying more negative charges due to the complex branching and frequent sialylation of their glycans (34). Usually, N-chain glycosylation events associated with a high positive charge in the V3 region disappear during tropism transition from CCR5 to CXCR4 strains (35). CXCR4 sequences of clusters 1 and 2 mostly lack N-linked glycosylation sites at the beginning of the V3 loop (positions 6 and 8), while cluster 4 deletions were mainly observed at positions 7 and 8. This demonstrated variance in co-receptor switching mechanisms within CRF01_AE clusters. Specifically, cluster 1 and 2 viruses are closer to the CRF01_AE ancestor strain based on the V3 sequence properties and structure, which are conducive to tropic switch. In contrast, the other sub-clusters favor CCR5 persistence to some extent, especially cluster 5.

CD4^+^ T-cell depletion and chronic inflammation are the signature processes in the development of HIV, driving the progression of the entire disease (23, 24). Following recent studies, both pyroptosis and apoptosis of CD4^+^ T cells are associated with the progression of HIV-1 infection. The death of these cells could result in a substantial release of IL-1β and IL-18, potentially exacerbating chronic inflammation (26, 36). In this investigation, we meticulously assessed the levels of cellular pyroptosis and apoptosis in early-stage HIV-1-infected individuals. The results unveiled a discernible upregulation in the expression of genes intricately involved in both processes. Particularly salient is the augmented incidence of cellular pyroptosis or apoptosis in individuals displaying compromised CD4^+^ T-cell counts (≤200) during the nascent phases of infection, hinting at a potential synergistic mechanism contributing to cellular depletion. Notably, the deposition of pyroptotic proteins and inflammatory factors establishes a pernicious pathogenic cycle. In this intricate cycle, dying CD4^+^ T cells emit inflammatory signals, drawing an increased cellular cohort to undergo demise within the infected tissues and amplifying inflammatory responses. Interestingly, intriguing is the revelation in our study of the distinctive features of highly pathogenic viruses (clusters 1 and 2). The elevated rate of conversion to CXCR4-tropic viruses within these clusters precipitates a swift depletion of CD4^+^ T cells in infected individuals. Therefore, the emergence of highly pathogenic CXCR4-tropic viruses and the initiation of chronic inflammation stand as hallmark processes in the pathogenesis of HIV, propelling the swift progression of the disease. Our current findings unveil potential unexpected connections between these two processes in individuals infected with highly pathogenic viruses. While the direct relationship between the two remains unclear, they also conspire to create a malignant pathogenic cycle, resulting in heightened cellular mortality.

In conclusion, CRF01_AE sub-clusters exhibit remarkably complex phenotypes and clinical manifestations. Differences in their genetic properties, protein modifications, and structural features determine the co-receptor selection preferences and suitability, as well as the virulence of viral clusters. Regularly monitoring HIV-1 genetic evolution and phenotypic changes is crucial to understanding how selection bias across risk groups and long-term therapy influence the balance between transmissible and toxic trade-offs.

## MATERIALS AND METHODS

### Participants and study design

This observational cohort study was conducted at the National Center for AIDS/STD Control and Prevention and constitutes the largest HIV molecular epidemiology survey in mainland China. A total of 7,196 HIV/AIDS patients were included in this study through data collected from the NHMES in mainland China. The data referred to 2,683 CRF01_AE, 3,347 CRF07_BC, and 1,166 sub-type B infections. All data from enrolled participants included HIV-1 sequence data and metadata such as transmission route, age at diagnosis, and baseline CD4^+^ T-cell count. Additionally, 74 cART-naïve individuals (cases) infected within 6 months were recruited for next-generation sequencing and primary virus isolation from two cohort studies: the Beijing Chaoyang District MSM cohort and the Guangxi Nanning observational cohort studies (Fig. S1).

### Relative reduction of CD4^+^ T-cell count in different cluster

To permit better direct comparison of individual CD4^+^ T count levels in infection, the *R*_T/F_ $\left(\frac{T_{healthy}-T}{T_{healthy}-T_{AIDS}}\times 100\right)$ indicator was used to quantify the relative decrease in CD4^+^ T counts due to transmitted/founder (*T*/*F*) virus (37). *T* corresponds to the absolute CD4^+^ T-cell count, and $T_{healthy}$ represents the pre-infection CD4^+^ T-cell count. Data from the CD4^+^ T-cell counts of healthy and uninfected adults in China were used in this study, with $T_{AIDS}$ = 200 cells/µL considered as the CD4^+^ T-cell count in the AIDS phase combined with other parameters described previously (37–39). *R*_T/F_ was considered a more reliable indicator of disease severity than absolute CD4^+^ T-cell counts, with higher values indicating relatively more virulent strains.

### RNA extraction and cDNA synthesis

Viral genomic RNA was extracted from 200 µL of plasma samples using the QIAmp Viral RNA Mini kit (Qiagen). The isolated RNA was then reverse transcribed using the Invitrogen SuperScript III reverse transcriptase kit (Thermo Fisher Scientific). Nested PCR was employed to amplify segments of the pol RT region (HXB2: nt2253–3554) and the env C2-C4 region (HXB2: nt7002–7541). Subsequent sequencing and sub-type consistency determination were carried out as previously described (13, 40).

### Determination of HIV-1 infection status

The HIV-1 limiting-antigen avidity enzyme-linked immunoassay kit (Beijing Kinghawk Pharmaceutical) was used to discriminate recent and long-term HIV-1 infection. Confirmation tests in triplicates were conducted for each sample. Samples with an ODn value of >1.5 were categorized as long-term HIV infections, whereas those with ODn values ranging from 0.4 to 1.5 were classified as recent HIV infections.

### Next-generation sequencing and analysis

The Illumina MiSeq library was prepared using a nested PCR approach. *env* C2–C5 (HXB2: nt6954–7668) genes were amplified in the first PCR round (13). The obtained PCR products were used in the second PCR round for amplification after cleaning using a MinElute PCR Purification Kit (Qiagen). Primers with 8-nt Illumina index and adapters added at both ends were used in the second PCR rounds (Fig. S4; Table S6). Second-round amplicons were purified by the KAPA PureBeads (Roche, Basel) and quantified using the KAPA SYBR FAST quantitative PCR (qPCR) kit (KAPA Biosystems) according to the manufacturer’s instructions. Prior to library sequencing, the 4-nM conjugate library was denatured with a sodium hydroxide solution and diluted with a hybridization buffer in the Miseq Reagent kit (Illumina). To increase library diversity, the denatured 40% PhiX control library (PhiX Control kit, version 3; Illumina) was added to each sample. The mixture was then added to the MiSeq Reagent kit and sequenced on the Illumina MiSeq system.

Raw data were processed and merged into fastq files using the FLASH software (41). The Galaxy server (https://usegalaxy.org/) was used for data quality control and filtering based on standard parameters (42). After trimming the primer region of each sample, samples with the same bases were combined into one haplogroup. The total frequency of each haplogroup was calculated, and the most common haplogroup was selected for further analysis. The most common haplotype in each sample was selected among all deep-sequenced individuals. To infer the phylogenetic relationship, a maximum-likelihood approach with the gamma distribution for rate heterogeneity GTR + G + I model was used in IQ-TREE (version 1.6.12) (43). The alternate topologies of whole sequencing data were estimated using the Shimodaira-Hasegawa test.

### Viral tropism prediction

To determine viral tropism, the V3 loop region was analyzed using Geno2pheno algorithm tools (https://coreceptor.geno2pheno.org/). Consistent with previous studies and to ensure the reliability of our results, the critical threshold for the FPR of Geno2pheno was set at 2% for delineating viral phenotypes. Samples with FPR values of ≤2% were designated as CXCR4 tropic or CCR5/CXCR4 (R5 × 4) dual tropic, while those with FPR values of >2% were classified as CCR5-tropic viruses (13, 44). The FPR value of each haplogroup obtained by next-generation sequencing was predicted for genotypic co-receptor. To ensure accuracy, only haplogroups with three or more occurrences of the V3 region within the same sample were used for prediction. The frequency distribution of FPR values for each sample was determined by analyzing the frequency of each V3 haplotype among the total number of reads.

### Primary virus isolation from peripheral blood mononuclear cells

PBMCs were isolated from peripheral blood by Ficoll-Paque (Amersham Biosciences, Uppsala, Sweden) density centrifugation and stored in liquid nitrogen. The primary virus was obtained by co-culturing 1 × 10^7^ PBMCs isolated from HIV-1 infected individuals with an equal number of phytohemagglutinin (PHA; Murex, Kent, UK)-stimulated PBMCs from HIV-1-negative blood donors. The cell mixture was cultured in RPMI-1640 medium containing 10% fetal bovine serum (FBS), 20-U recombinant IL-2 (PeproTech), and 1% penicillin/streptomycin (Sigma-Aldrich, Milan, Italy) (45, 46), and maintained for 4 weeks with regular removal of half of the cell culture supernatant every 3 days and supplementation with fresh medium. Half of the entire cell mixture was discarded and replaced with fresh 5 × 10^6^ PHA-stimulated PBMCs from HIV-negative subjects once a week. Viral isolation was monitored twice a week (on days 4, 7, 10, 14, 17, 21, and 24), and the p24 antigen level in the culture supernatant was measured using a commercially available enzyme-linked immunosorbent assay (ELISA) kit to determine the growth dynamics of the primary virus. Cultures were considered positive for viral isolation when the p24 antigen level exceeded the threshold value in two consecutive cultures.

### Identification of co-receptor usage

To identify co-receptor usage, GHOST-CXCR4 and GHOST-CCR5 cell lines were generated from the human osteosarcoma cell line and stably expressed CD4 along with chemokine receptors CXCR4 or CCR5 (47, 48). GHOST cells were seeded at a density of 6 × 10^4^ cells per well in 24-well plates (Corning Incorporated). After 24 h, the medium was removed, and undiluted primary viral isolates were added, along with 8-µg/mL diethyl-aminoethyl-dextran (DEAE) to enhance infection efficiency. After 5 h of culture at 37°C, infected cells were washed with Dulbecco's modified Eagle medium (DMEM ) to remove residual viruses and then supplemented with DMEM containing 10% FBS at 37°C. Infected GHOST cells were cultured for 48 h, and GFP expression was assessed using a fluorescence microscope or a flow cytometer (BD FACSCalibur). Briefly, 15,000–20,000 cells were examined and collected, and the utilization of HIV-1 viral co-receptors was determined based on the rate of GFP expression. Generally, the average GFP fluorescence in infected cells is expected to undergo an approximately 2.5-fold change compared to uninfected cells (46). GHOST-CCR5 and GHOST-CXCR4 cells were used as positive controls, with XJ13 and NL4-3 strains, respectively, while cells not infected with HIV-1 served as negative controls in each experiment (49). To validate the co-receptor utilization of the viruses, we also employed the CCR5 inhibitor Maraviroc (Sigma-Aldrich) and the CXCR4 inhibitor AMD3100 (Sigma-Aldrich). Treatment of GHOST cells with these antagonists occurred 1 h prior to infection, with final concentrations set at 2 nM and 2 µM.

### Sequence variation analysis and structural model building

Sequences were aligned using the MUSCLE (version 3.8.4). The ProtParam online tool (https://web.expasy.org/protparam/) was used to determine the length of amino acids and the number of charges in amino acid residues. To illustrate the characteristics of the V3 loop in each sub-type, a consensus nucleotide sequence was generated for each cluster using WebLogo (http://weblogo.threeplusone.com/). Additionally, to investigate the divergences in the dynamics of *env*-V3 sequences in different clusters, sequence divergence was calculated as the pairwise genetic distance (number of nucleotide substitutions per locus) between cluster sequence variants and the consensus sequence of the ancestral Thailand strain using TBtools (version 5.4.5) (50) and Entropy (https://www.hiv.lanl.gov/content/sequence/ENTROPY/entropy.html).

The initial tertiary structure of HIV *env*-V3 was modeled using the SWISS-MODEL protein homology prediction tool, with the protein structure of *env*-gp120 (PDB ID: 6NQD) serving as the template. The previous CXCR4 structure was also used to generate the initial template for the CXCR4-V3 protein complex docking model (51). Finally, analysis and identification of interactions and positions with signature variations were performed using PyMOL (version 2.5.0).

### Real-time qPCR

Total RNA was extracted from infected PBMCs using the miRNeasy Mini Kit (Qiagen), and RNA was transcribed using PrimpScript RT-PCR Kit (TAKARA) according to the manufacturer’s instructions. qPCR was performed using 2 x SYBR Premix Ex TaqTM II (TAKARA). The ABI Prism 7900HT (Applied Biosystems) was used for qPCR reactions, and GAPDH was used as the internal reference (52). Fold changes in gene expression were calculated using the 2-ΔΔCt method.

### IL-1β and IL-18 release assay

The levels of human IL-1β and IL-18 in plasma were measured using an ELISA Kit (Elabscience, Wuhan, China) following the manufacturer’s instruction. IL-1β and IL-18 levels were expressed as picogram per milliliter.

### Statistics

The data were analyzed using Statistical Package for the Social Sciences (version 24.0 (IBM, Chicago, IL, USA). The association of virus tropisms within various CRF01_AE clusters was assessed through Pearson’s χ test and Cochran-Armitage trend test. Differences in proportions or rates between two groups were evaluated using the Mann–Whitney *U* test or Fisher’s exact test. The linear relationship between two factors was determined based on Pearson’s correlation coefficients. All tests were two-sided (*α* = 0.05), and differences were statistically significant if the *P* value was <0.05.

## Data Availability

The relevant data that support the findings of this study are available on request from the corresponding author. The sequencing data from the HIV-positive individuals are deposited in the National Center for Biotechnology Information GenBank under accession numbers PP098653–PP098724 and MH672692–MH673032. The protein structure data are available at the PDB under ID 6NQD and in reference 51. Remaining data are found in the supplemental material.

## SUPPLEMENTAL MATERIAL

Supplemental figures and tables mbio.03349-23-s0001.docx Fig. S1 to S4; Tables S1 to S6.

ASM does not own the copyrights to Supplemental Material that may be linked to, or accessed through, an article. The authors have granted ASM a non-exclusive, world-wide license to publish the Supplemental Material files. Please contact the corresponding author directly for reuse.
