# Supplementary material for: Distinct genetic clusters in HIV-1 CRF01_AE-infected patients induced variable degrees of CD4+ T-cell loss
Source: mBio. 2024 Feb 22;15(3):e03349-23. doi: 10.1128/mbio.03349-23 (PMC10936439; doi:10.1128/mbio.03349-23)
Supplement: Supplemental figures and tables — Fig. S1 to S4; Tables S1 to S6. [file mbio.03349-23-s0001.docx]

**This file includes:**

Figs. S1 to S4

Tables S1 to S6

**
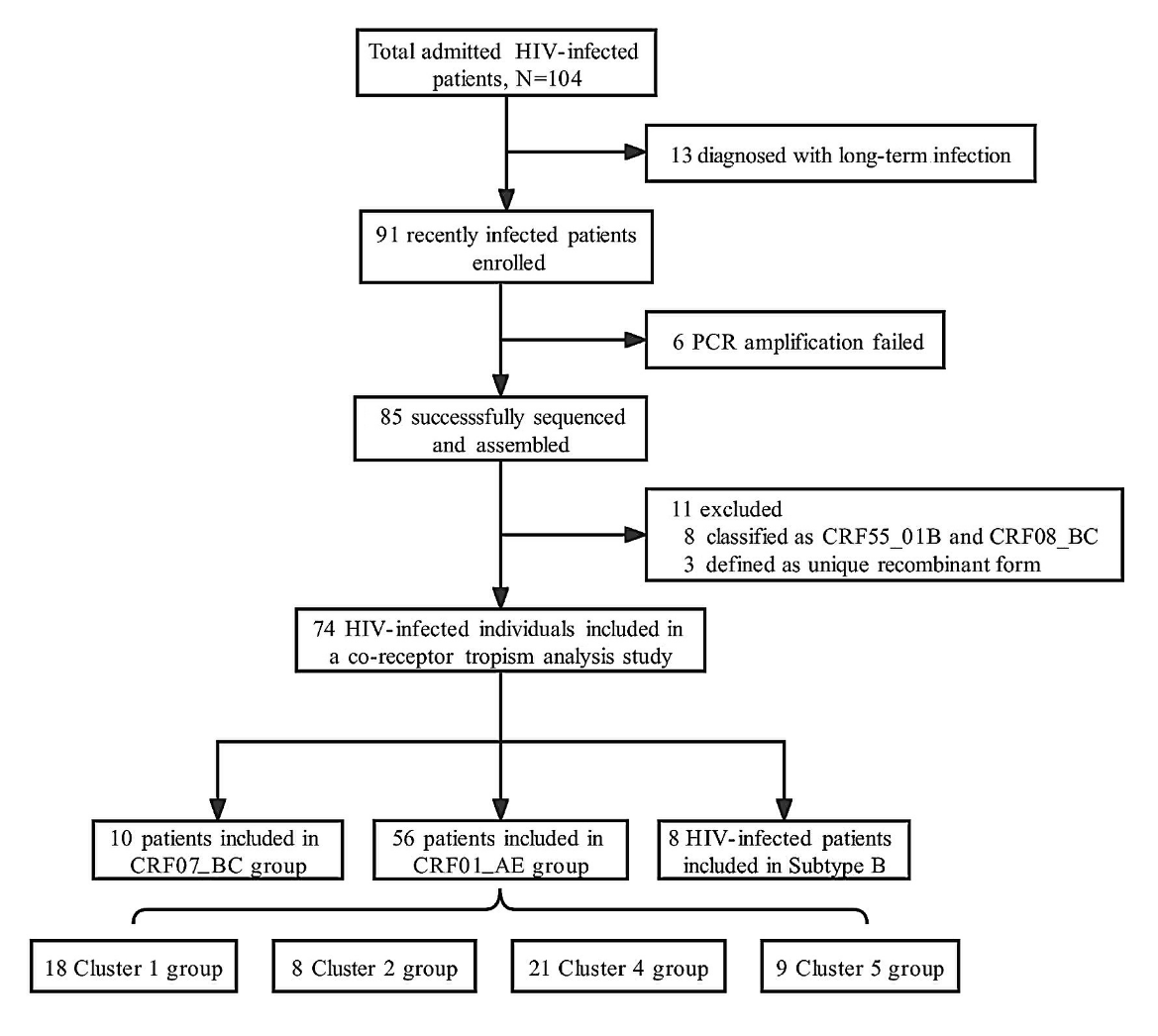
**

**Fig. S1.** Flowchart of the filtration process for study participants. Out of the initial sample set, 74 samples successfully passed the criteria, including 56 of pure CRF01_AE, 10 samples of CRF07_BC, and 8 samples of subtype B infections. Samples from this selected population underwent next-generation sequencing and further analysis for coreceptor tropism.


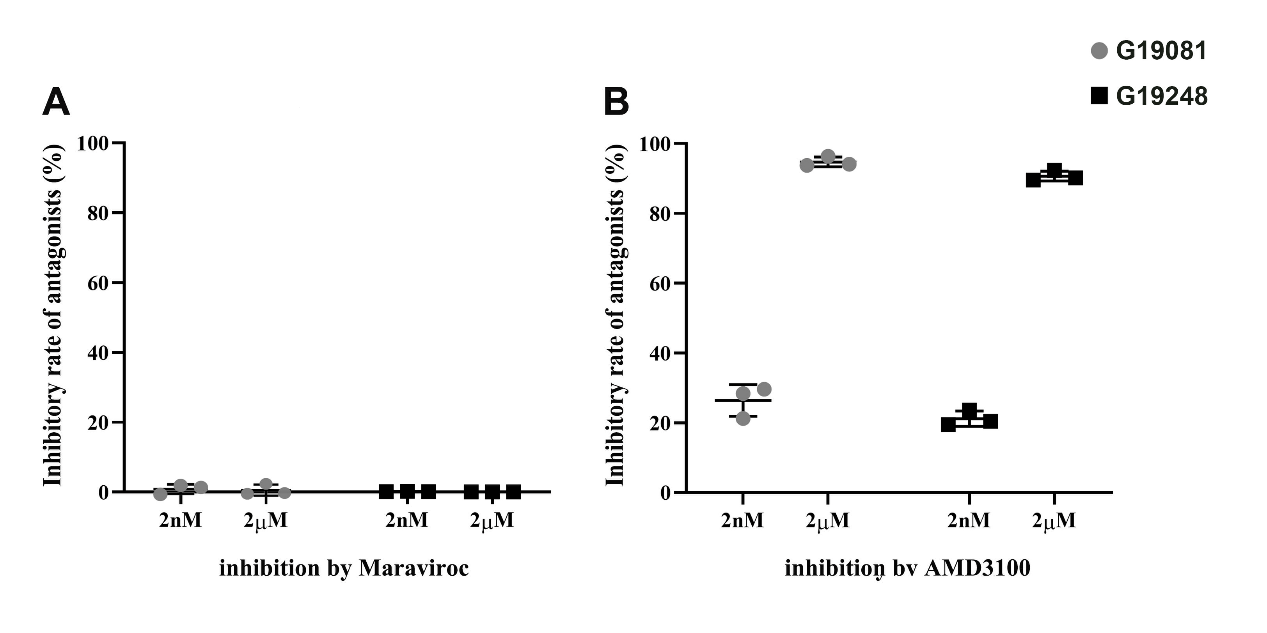


**Fig. S2.** Determination of viral tropism using coreceptor blockers. (A) Inhibitory effect of maraviroc on viruses. (B) Inhibitory effect of AMD3100 on viruses. GHOST cells were treated with either AMD3100 or maraviroc, respectively, and GFP expression was assessed in a flow cytometer (BD FACSCalibur). Approximately 20,000 cells were examined and collected during this process. The inhibitory rate (%) was calculated as follows: (1 – number of infected cells in the presence of drugs/ number of infected cells in the absence of drugs)×100. The mean inhibitory rate is shown with standard deviations based on three independent measurements.


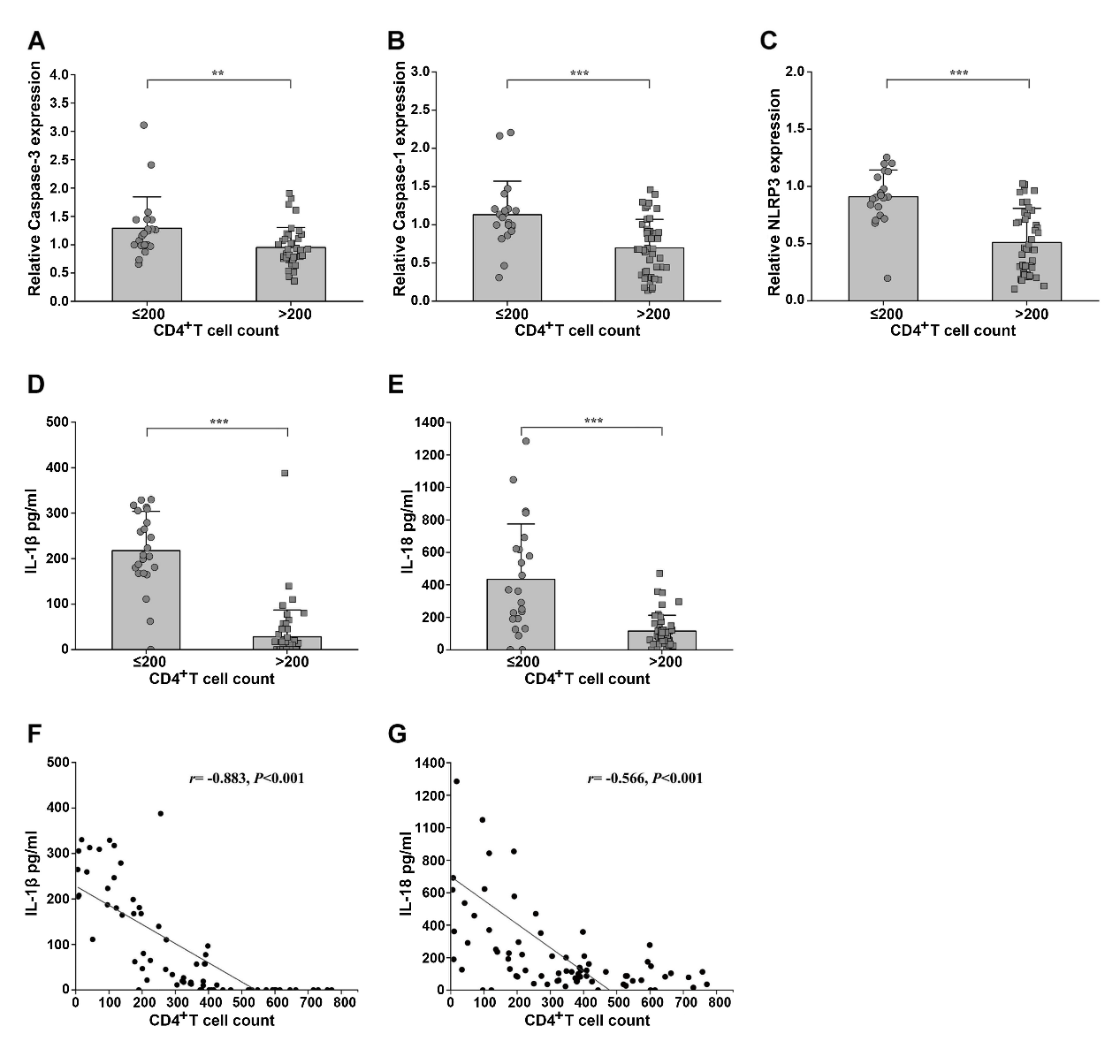


**Fig. S3.** Activation of inflammasome and expression of plasma IL-1β and IL-18. (A-C) Quantitative expression of genes coding for NLRP3, Caspase-1 and Caspase-3 in peripheral blood mononuclear cells (PBMCs) from each patient in the low (≤ 200) CD4+ T cell count group and the high (> 200) CD4^+^ T cell count group. Mean values and standard error of the mean (SEM) are shown. Fold change was determined based on the 2-ΔΔCt method. (C–D) Release of IL-1β and IL-18 in the plasma of each patient in the low (≤ 200) CD4+ T cell count group and the high (> 200) CD4+ T cell count group. (E–F) Correlation between IL-1β and IL-18 levels and CD4+ T cell counts. ***, ** and * indicate, *P* < 0.001, *P* < 0.01 and *P* < 0.05, respectively. *R*, correlation coefficient.


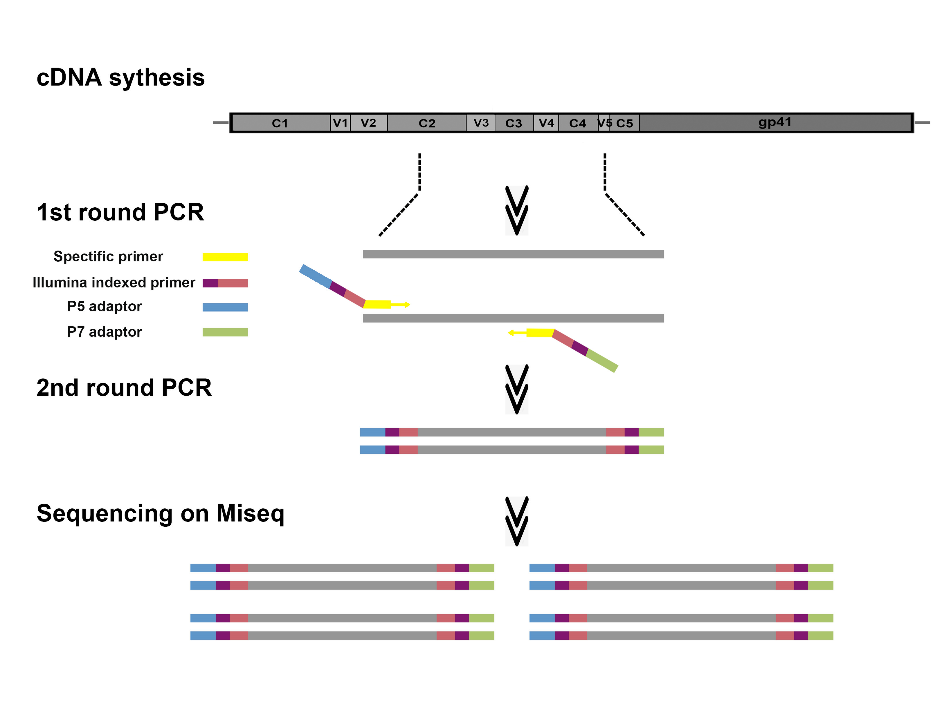


**Fig. S4.** Design principle of Illumina MiSeq for next-generation sequencing. The C2V3 region in the *env* gene of HIV was amplified using a nested PCR approach. Primers used in the second PCR round were modified to include sample indexes and Illumina adapters (P5 and P7). The resulting library was then sequenced on an Illumina MiSeq platform.

| **Table S1.** Prevalence of predicted CXCR4 viruses in the low (≤ 200) and high (> 200) CD4^+^ T cell count groups within individuals infected with the HIV-1 CRF01_AE subtype. | | | |
| --- | --- | --- | --- |
|  | CD4^+^ cell count ≤ 200  (N=308) | CD4^+^ cell count > 200  (N=1178) | *P*-value |
|  |  |  |  |
| Coreceptor usage, N (%) |  |  | <0.05 |
| CXCR4 (FPR ≤ 2%) | 159(51.6) | 132(11.2) |  |
| CCR5 (FPR > 2%) | 149 (48.4) | 1046(88.8) |  |
| The *P-*value was calculated using the chi-square test. | | | |

| **Table S2.** Comparison of the low (≤ 200) and high (> 200) CD4^+^ T cell count groups within HIV-1-infected patients with different CRF01_AE clusters. | | | | | |
| --- | --- | --- | --- | --- | --- |
|  | Cluster 1  (N=407) | Cluster 2 (N=248) | Cluster 4  (N =539) | Cluster 5 (N=325) | *P*-value |
| CD4^+^ T cell count groups, N (%) |  |  |  |  | <0.01 |
| CD4^+^ T cell count ≤ 200 (cells/μL） | 138(33.9) | 72(29.0) | 56(10.4) | 7(2.2) |  |
| CD4^+^ T cell count > 200 (cells/μL） | 269(66.1) | 176(71.0) | 483(89.6) | 318(97.8) |  |
| The *P*-value was calculated using the Cochran-Armitage trend test. | | | | | |

| **Table S3.** Prevalence of predicted HIV-1 CXCR4 viruses in different CRF01_AE clusters. | | | | | |
| --- | --- | --- | --- | --- | --- |
|  | Cluster 1  (N=521) | Cluster 2 (N=249) | Cluster 4  (N =597) | Cluster 5 (N=341) | *P*-value |
| Coreceptor usage, N (%) |  |  |  |  | <0.01 |
| CXCR4 (FPR ≤ 2%) | 115(22.1) | 51(20.5) | 91(15.2) | 21(6.2) |  |
| CCR5 (FPR > 2%) | 406(77.9) | 198(79.5) | 506(84.8) | 320(93.8) |  |
| The *P*-value was calculated using the Cochran-Armitage trend test. | | | | | |

| **Table S4.** Baseline characteristics of HIV-1-infected participants enrolled in the study. | | | | |
| --- | --- | --- | --- | --- |
| Sample number | Age | Gender | Baseline CD4^+^ cell count (cells/μL) | Route of HIV transmission |
| G19013 | 52 | Male | 731 | Heterosexual |
| G19069 | 33 | Male | 18 | Heterosexual |
| G19070 | 59 | Female | 190 | Heterosexual |
| G19074 | 29 | Female | 215 | Heterosexual |
| G19075 | 41 | Male | 95 | Heterosexual |
| G19077 | 43 | Female | 574 | Heterosexual |
| G19078 | 56 | Male | 396 | Heterosexual |
| G19079 | 43 | Female | 293 | Heterosexual |
| G19080 | 43 | Male | 259 | Heterosexual |
| G19081 | 40 | Male | 116 | Heterosexual |
| G19086 | 66 | Female | 392 | Heterosexual |
| G19092 | 45 | Female | 534 | Heterosexual |
| G19093 | 75 | Male | 175 | Heterosexual |
| G19099 | 47 | Male | 271 | Heterosexual |
| G19100 | 34 | Female | 142 | Heterosexual |
| G19104 | 61 | Male | 71 | Heterosexual |
| G19105 | 32 | Male | 86 | Heterosexual |
| G19106 | 47 | Male | 140 | Heterosexual |
| G19002 | 39 | Male | 191 | Heterosexual |
| G19004 | 59 | Male | 195 | Heterosexual |
| G19028 | 30 | Female | 443 | Heterosexual |
| G19049 | 53 | Male | 375 | Heterosexual |
| G19066 | 39 | Male | 531 | Heterosexual |
| G19067 | 52 | Male | 96 | Heterosexual |
| G19072 | 30 | Female | 147 | Heterosexual |
| G19098 | 74 | Male | 398 | Heterosexual |
| G19171 | 23 | Male | 201 | Male-to-male sexual |
| G19176 | 25 | Male | 178 | Male-to-male sexual |
| G19178 | 40 | Male | 305 | Male-to-male sexual |
| G19180 | 22 | Male | 663 | Male-to-male sexual |
| G19182 | 24 | Male | 425 | Male-to-male sexual |
| G19194 | 24 | Male | 364 | Male-to-male sexual |
| G19222 | 26 | Male | 582 | Male-to-male sexual |
| G19223 | 26 | Male | 758 | Male-to-male sexual |
| G19240 | 29 | Male | 384 | Male-to-male sexual |
| G19243 | 23 | Male | 548 | Male-to-male sexual |
| G19245 | 22 | Male | 136 | Male-to-male sexual |
| G19248 | 39 | Male | 10 | Male-to-male sexual |
| G19249 | 25 | Male | 409 | Male-to-male sexual |
| G19252 | 39 | Male | 467 | Male-to-male sexual |
| G19253 | 35 | Male | 291 | Male-to-male sexual |
| G19259 | 29 | Male | 599 | Male-to-male sexual |
| G19270 | 41 | Male | 18 | Male-to-male sexual |
| G19279 | 24 | Male | 402 | Male-to-male sexual |
| G19280 | 30 | Male | 347 | Male-to-male sexual |
| G19286 | 25 | Male | 593 | Male-to-male sexual |
| G19287 | 22 | Male | 409 | Male-to-male sexual |
| G19167 | 26 | Male | 527 | Male-to-male sexual |
| G19179 | 30 | Male | 197 | Male-to-male sexual |
| G19186 | 22 | Male | 381 | Male-to-male sexual |
| G19192 | 23 | Male | 348 | Male-to-male sexual |
| G19206 | 20 | Male | 603 | Male-to-male sexual |
| G19215 | 23 | Male | 416 | Male-to-male sexual |
| G19250 | 36 | Male | 716 | Male-to-male sexual |
| G19255 | 42 | Male | 325 | Male-to-male sexual |
| G19293 | 33 | Male | 615 | Male-to-male sexual |
| G19175 | 23 | Male | 282 | Male-to-male sexual |
| G19187 | 22 | Male | 211 | Heterosexual |
| G19190 | 19 | Male | 318 | Male-to-male sexual |
| G19191 | 45 | Male | 574 | Male-to-male sexual |
| G19198 | 41 | Male | 333 | Male-to-male sexual |
| G19263 | 20 | Male | 324 | Male-to-male sexual |
| G19274 | 22 | Male | 384 | Male-to-male sexual |
| G19292 | 32 | Male | 380 | Male-to-male sexual |
| G19034 | 32 | Male | 345 | Heterosexual |
| G19036 | 28 | Male | 6 | Male-to-male sexual |
| G19038 | 50 | Male | 602 | Heterosexual |
| G19064 | 46 | Male | 42 | Heterosexual |
| G19071 | 45 | Male | 384 | Heterosexual |
| G19083 | 37 | Male | 390 | Heterosexual |
| G19084 | 23 | Male | 528 | Male-to-male sexual |
| G19090 | 67 | Male | 116 | Heterosexual |
| G19102 | 34 | Male | 51 | Heterosexual |
| G19103 | 51 | Male | 71 | Heterosexual |

| **Table S5.** Total number of next-generation sequencing reads obtained for each sample and the frequency distribution of Geno2pheno false positive rate (FPR) values. | | | | | | | |
| --- | --- | --- | --- | --- | --- | --- | --- |
| Sample number | Viral subtype | Number of clean reads | Reads in each Geno2pheno FPR interval (%) | | | | |
|  |  |  | < 2% | 2–5% | 5–10% | 10–15% | > 15% |
| G19013 | CRF01_AE Cluster1 | 9121 | 63.3 | 34.4 | 2.3 | 0.0 | 0.0 |
| G19069 | CRF01_AE Cluster1 | 18351 | 46.2 | 13.1 | 40.7 | 0.0 | 0.0 |
| G19070 | CRF01_AE Cluster1 | 75108 | 8.4 | 0.0 | 0.0 | 0.0 | 91.6 |
| G19074 | CRF01_AE Cluster1 | 97464 | 0.0 | 0.0 | 0.7 | 0.0 | 99.3 |
| G19075 | CRF01_AE Cluster1 | 38322 | 13.8 | 0.0 | 6.9 | 79.3 | 0.0 |
| G19077 | CRF01_AE Cluster1 | 41630 | 9.0 | 89.3 | 1.0 | 0.2 | 0.5 |
| G19078 | CRF01_AE Cluster1 | 73303 | 18.9 | 35.5 | 8.4 | 29.1 | 8.1 |
| G19079 | CRF01_AE Cluster1 | 68344 | 1.8 | 0.8 | 36.2 | 0.0 | 61.2 |
| G19080 | CRF01_AE Cluster1 | 117252 | 0.0 | 0.0 | 0.0 | 0.1 | 99.9 |
| G19081 | CRF01_AE Cluster1 | 7652 | 13.6 | 86.4 | 0.0 | 0.0 | 0.0 |
| G19086 | CRF01_AE Cluster1 | 91028 | 2.4 | 97.6 | 0.0 | 0.0 | 0.0 |
| G19092 | CRF01_AE Cluster1 | 64384 | 9.7 | 0.0 | 1.2 | 3.2 | 85.9 |
| G19093 | CRF01_AE Cluster1 | 22428 | 99.5 | 0.5 | 0.0 | 0.0 | 0.0 |
| G19099 | CRF01_AE Cluster1 | 34991 | 10.1 | 0.0 | 0.0 | 0.0 | 89.9 |
| G19100 | CRF01_AE Cluster1 | 19521 | 47.9 | 13.1 | 39.1 | 0.0 | 0.0 |
| G19104 | CRF01_AE Cluster1 | 31228 | 15.4 | 52.9 | 26.6 | 1.4 | 3.8 |
| G19105 | CRF01_AE Cluster1 | 52116 | 97.5 | 1.8 | 0.7 | 0.0 | 0.0 |
| G19106 | CRF01_AE Cluster1 | 75974 | 6.2 | 80.2 | 1.8 | 9.5 | 2.3 |
| G19002 | CRF01_AE Cluster2 | 22650 | 3.4 | 96.6 | 0.0 | 0.0 | 0.0 |
| G19004 | CRF01_AE Cluster2 | 86210 | 0.0 | 0.0 | 0.0 | 0.0 | 100.0 |
| G19028 | CRF01_AE Cluster2 | 51090 | 0.0 | 0.0 | 30.4 | 0.0 | 69.6 |
| G19049 | CRF01_AE Cluster2 | 65480 | 0.0 | 0.0 | 0.0 | 0.0 | 100 |
| G19066 | CRF01_AE Cluster2 | 45220 | 0.0 | 0.0 | 89.7 | 10.3 | 0.0 |
| G19067 | CRF01_AE Cluster2 | 71760 | 77.7 | 22.4 | 0.0 | 0.0 | 0.0 |
| G19072 | CRF01_AE Cluster2 | 56390 | 3.3 | 32.6 | 62.8 | 1.2 | 0.1 |
| G19098 | CRF01_AE Cluster2 | 66390 | 18.4 | 20.6 | 42.3 | 16.0 | 2.7 |
| G19171 | CRF01_AE Cluster4 | 7020 | 0.0 | 0.1 | 2.2 | 1.3 | 96.4 |
| G19176 | CRF01_AE Cluster4 | 4242 | 32.3 | 67.3 | 0.2 | 0.0 | 0.2 |
| G19178 | CRF01_AE Cluster4 | 5912 | 0.0 | 1.5 | 5.3 | 90.8 | 2.4 |
| G19180 | CRF01_AE Cluster4 | 51190 | 4.2 | 94.9 | 0.7 | 0.0 | 0.3 |
| G19182 | CRF01_AE Cluster4 | 60069 | 3.6 | 92.0 | 2.8 | 0.0 | 1.6 |
| G19194 | CRF01_AE Cluster4 | 63235 | 77.2 | 5.4 | 15.9 | 0.2 | 1.4 |
| G19222 | CRF01_AE Cluster4 | 83649 | 0.0 | 1.3 | 42.7 | 2.9 | 53.0 |
| G19223 | CRF01_AE Cluster4 | 74391 | 5.4 | 70.6 | 23.4 | 0.4 | 0.2 |
| G19240 | CRF01_AE Cluster4 | 57749 | 4.4 | 95.1 | 0.5 | 0.0 | 0.0 |
| G19243 | CRF01_AE Cluster4 | 47790 | 0.1 | 3.5 | 69.3 | 4.9 | 22.3 |
| G19245 | CRF01_AE Cluster4 | 28844 | 26.2 | 72.7 | 1.1 | 0.0 | 0.0 |
| G19248 | CRF01_AE Cluster4 | 170248 | 8.0 | 0.8 | 1.5 | 1.5 | 88.1 |
| G19249 | CRF01_AE Cluster4 | 124992 | 0.4 | 0.1 | 1.4 | 2.3 | 95.8 |
| G19252 | CRF01_AE Cluster4 | 84423 | 0.1 | 21.9 | 76.7 | 1.0 | 0.3 |
| G19253 | CRF01_AE Cluster4 | 43377 | 6.0 | 93.8 | 0.2 | 0.0 | 0.0 |
| G19259 | CRF01_AE Cluster4 | 54511 | 0.0 | 0.1 | 2.9 | 1.8 | 95.1 |
| G19270 | CRF01_AE Cluster4 | 97402 | 20.3 | 19.9 | 22.2 | 0.7 | 36.9 |
| G19279 | CRF01_AE Cluster4 | 90494 | 4.0 | 95.4 | 0.5 | 0.0 | 0.0 |
| G19280 | CRF01_AE Cluster4 | 87421 | 0.3 | 7.6 | 87.9 | 2.8 | 1.4 |
| G19286 | CRF01_AE Cluster4 | 63606 | 3.5 | 94.9 | 1.3 | 0.4 | 0.0 |
| G19287 | CRF01_AE Cluster4 | 55014 | 98.6 | 1.3 | 0.1 | 0.0 | 0.0 |
| G19167 | CRF01_AE Cluster5 | 9204 | 0.0 | 0.0 | 0.9 | 1.1 | 98.0 |
| G19179 | CRF01_AE Cluster5 | 5092 | 1.4 | 1.3 | 22.4 | 0.9 | 73.9 |
| G19186 | CRF01_AE Cluster5 | 36267 | 0.0 | 0.3 | 1.8 | 1.9 | 95.9 |
| G19192 | CRF01_AE Cluster5 | 90899 | 0.0 | 0.0 | 0.0 | 0.0 | 100.0 |
| G19206 | CRF01_AE Cluster5 | 70983 | 0.0 | 1.2 | 4.1 | 2.8 | 91.8 |
| G19215 | CRF01_AE Cluster5 | 72970 | 0.0 | 0.0 | 0.1 | 0.4 | 99.6 |
| G19250 | CRF01_AE Cluster5 | 54321 | 0.0 | 1.0 | 5.5 | 90.5 | 2.9 |
| G19255 | CRF01_AE Cluster5 | 66925 | 0.0 | 0.0 | 0.2 | 0.1 | 99.8 |
| G19293 | CRF01_AE Cluster5 | 20228 | 0.0 | 0.0 | 1.5 | 0.6 | 97.9 |
| G19175 | B | 2924 | 0.0 | 0.0 | 0.0 | 0.0 | 100.0 |
| G19187 | B | 51577 | 0.0 | 1.5 | 4.0 | 90.3 | 4.2 |
| G19190 | B | 47548 | 0.0 | 0.0 | 0.0 | 0.6 | 99.4 |
| G19191 | B | 54687 | 0.0 | 0.1 | 1.2 | 1.0 | 97.7 |
| G19198 | B | 74570 | 0.2 | 0.4 | 6.3 | 0.3 | 92.9 |
| G19263 | B | 66111 | 0.0 | 0.0 | 0.0 | 0.2 | 99.8 |
| G19274 | B | 64701 | 0.0 | 0.0 | 0.0 | 0.1 | 99.9 |
| G19292 | B | 142813 | 0.8 | 93.4 | 5.0 | 0.8 | 0.0 |
| G19034 | CRF07_BC | 87500 | 0.0 | 0.0 | 0.0 | 0.0 | 100.0 |
| G19036 | CRF07_BC | 52180 | 0.0 | 0.0 | 0.0 | 0.0 | 100.0 |
| G19038 | CRF07_BC | 37950 | 0.0 | 0.0 | 0.0 | 0.0 | 100.0 |
| G19064 | CRF07_BC | 49860 | 0.0 | 0.0 | 0.0 | 0.0 | 100.0 |
| G19071 | CRF07_BC | 40580 | 0.0 | 0.0 | 0.0 | 0.0 | 100.0 |
| G19083 | CRF07_BC | 102300 | 0.0 | 0.0 | 0.0 | 0.0 | 100.0 |
| G19084 | CRF07_BC | 35760 | 0.0 | 0.0 | 0.0 | 0.0 | 100.0 |
| G19090 | CRF07_BC | 72500 | 0.0 | 0.0 | 0.0 | 0.68 | 99.32 |
| G19102 | CRF07_BC | 44880 | 0.0 | 0.0 | 0.0 | 0.0 | 100.0 |
| G19103 | CRF07_BC | 56160 | 0.0 | 0.0 | 0.0 | 0.0 | 100.0 |

| **Table S6.** Primers used in second-round PCR amplifications in the nested PCR approach adopted in the present study for Illumina MiSeq library preparation. | | |
| --- | --- | --- |
| Forward | C2V3-P5-1 | 5'-AATGATACGGCGACCACCGAGATCTACACTAGATCGCTCGTCGGCAGCGTCAGATGTGTATAAGAGACAGCAGTACAATGYACACATGG-3’ |
|  | C2V3-P5-2 | 5'-AATGATACGGCGACCACCGAGATCTACACCTCTCTATTCGTCGGCAGCGTCAGATGTGTATAAGAGACAGCAGTACAATGYACACATGG-3’ |
|  | C2V3-P5-3 | 5'-AATGATACGGCGACCACCGAGATCTACACTATCCTCTTCGTCGGCAGCGTCAGATGTGTATAAGAGACAGCAGTACAATGYACACATGG-3’ |
|  | C2V3-P5-4 | 5'-AATGATACGGCGACCACCGAGATCTACACAGAGTAGATCGTCGGCAGCGTCAGATGTGTATAAGAGACAGCAGTACAATGYACACATGG-3’ |
|  | C2V3-P5-5 | 5'-AATGATACGGCGACCACCGAGATCTACACGTAAGGAGTCGTCGGCAGCGTCAGATGTGTATAAGAGACAGCAGTACAATGYACACATGG-3’ |
|  | C2V3-P5-6 | 5'-AATGATACGGCGACCACCGAGATCTACACACTGCATATCGTCGGCAGCGTCAGATGTGTATAAGAGACAGCAGTACAATGYACACATGG-3’ |
|  | C2V3-P5-7 | 5'-AATGATACGGCGACCACCGAGATCTACACAAGGAGTATCGTCGGCAGCGTCAGATGTGTATAAGAGACAGCAGTACAATGYACACATGG-3’ |
|  | C2V3-P5-8 | 5'-AATGATACGGCGACCACCGAGATCTACACCTAAGCCTTCGTCGGCAGCGTCAGATGTGTATAAGAGACAGCAGTACAATGYACACATGG-3’ |
| Reverse | C2V3-P7-1 | 5'-CAAGCAGAAGACGGCATACGAGATTCGCCTTAGTCTCGTGGGCTCGGAGATGTGTATAAGAGACAGAGAAAAATTCYCCTCYACAA-3’ |
|  | C2V3-P7-2 | 5'-CAAGCAGAAGACGGCATACGAGATCTAGTACGGTCTCGTGGGCTCGGAGATGTGTATAAGAGACAGAGAAAAATTCYCCTCYACAA-3’ |
|  | C2V3-P7-3 | 5'-CAAGCAGAAGACGGCATACGAGATTTCTGCCTGTCTCGTGGGCTCGGAGATGTGTATAAGAGACAGAGAAAAATTCYCCTCYACAA-3’ |
|  | C2V3-P7-4 | 5'-CAAGCAGAAGACGGCATACGAGATGCTCAGGAGTCTCGTGGGCTCGGAGATGTGTATAAGAGACAGAGAAAAATTCYCCTCYACAA-3’ |
|  | C2V3-P7-5 | 5'-CAAGCAGAAGACGGCATACGAGATAGGAGTCCGTCTCGTGGGCTCGGAGATGTGTATAAGAGACAGAGAAAAATTCYCCTCYACAA-3’ |
|  | C2V3-P7-6 | 5'-CAAGCAGAAGACGGCATACGAGATCATGCCTAGTCTCGTGGGCTCGGAGATGTGTATAAGAGACAGAGAAAAATTCYCCTCYACAA-3’ |
|  | C2V3-P7-7 | 5'-CAAGCAGAAGACGGCATACGAGATGTAGAGAGGTCTCGTGGGCTCGGAGATGTGTATAAGAGACAGAGAAAAATTCYCCTCYACAA-3’ |
|  | C2V3-P7-8 | 5'-CAAGCAGAAGACGGCATACGAGATCCTCTCTGGTCTCGTGGGCTCGGAGATGTGTATAAGAGACAGAGAAAAATTCYCCTCYACAA-3’ |
|  | C2V3-P7-9 | 5'-CAAGCAGAAGACGGCATACGAGATAGCGTAGCGTCTCGTGGGCTCGGAGATGTGTATAAGAGACAGAGAAAAATTCYCCTCYACAA-3’ |
|  | C2V3-P7-10 | 5'-CAAGCAGAAGACGGCATACGAGATCAGCCTCGGTCTCGTGGGCTCGGAGATGTGTATAAGAGACAGAGAAAAATTCYCCTCYACAA-3’ |
|  | C2V3-P7-11 | 5'-CAAGCAGAAGACGGCATACGAGATTGCCTCTTGTCTCGTGGGCTCGGAGATGTGTATAAGAGACAGAGAAAAATTCYCCTCYACAA-3’ |
|  | C2V3-P7-12 | 5'-CAAGCAGAAGACGGCATACGAGATTCCTCTACGTCTCGTGGGCTCGGAGATGTGTATAAGAGACAGAGAAAAATTCYCCTCYACAA-3’ |
|  | C2V3-P7-12 | 5'-CAAGCAGAAGACGGCATACGAGATTCCTCTACGTCTCGTGGGCTCGGAGATGTGTATAAGAGACAGAGAAAAATTCYCCTCYACAA-3’ |
